# Mixed *Leptospira* infections in domestic animals from a rural community with high leptospirosis endemicity

**DOI:** 10.1371/journal.pone.0312556

**Published:** 2024-10-29

**Authors:** Pamela Mosquera, Lorena Mejia, Gabriela Ortiz, Giuliana Pazmino, Talima Pearson, Verónica Barragán, Gabriel Trueba

**Affiliations:** 1 Instituto de Microbiología, Colegio de Ciencias Biológicas y Ambientales, Universidad San Francisco de Quito USFQ, Quito, Ecuador; 2 Pathogen and Microbiome Institute, Northern Arizona University, Flagstaff, Arizona, United States of America; UFPL, BRAZIL

## Abstract

**Background:**

Leptospirosis is one of the most common zoonoses in the world which is associated with a severe febrile disease in humans causing a variety of syndromes including meningitis, interstitial nephritis, hepatitis, and sometimes death. Leptospirosis is caused by different pathogenic *Leptospira* species divided into almost 30 serogroups and more than 300 serovars which are carried by some animal asymptomatic chronic infections. Humans can become infected through direct contact with animal urine or indirectly by coming into contact with fresh water or mud contaminated with urine.

**Methodology/Principal findings:**

In this research, we looked for leptospiral DNA in urine samples from dogs living in a rural, low-income and highly endemic community in the coast of Ecuador. We used molecular biology and next-generation sequencing for the detection. Our results showed evidence of two *Leptospira* species, *L interrogans* and *L*. *santarosai*, genomes in three dogs.

**Conclusions/Significance:**

It has been widely known that animal carriers are typically infected with a single leptospiral strain. However, recent reports, including the present one, indicate that carrier animals may be coinfected with two or more leptospiral species.

## Introduction

Leptospirosis is a worldwide zoonosis affecting more than 1 million people and close to 60.000 deaths per year [1]. Humans can become infected through direct contact with urine or tissues from infected animals, or indirectly by contacting water or soil that has been contaminated by urine [2].

Animal reservoirs (dogs, rats, pigs, etc.) are thought to suffer from chronic, asymptomatic kidney infections with prolonged leptospiral shedding via urine [2]; however, little is known about these long-lasting infections. One important feature of some chronic infections may be an increased likelihood of mixed infections. Recent reports show that some wildlife reservoirs suffer from mixed infections with more than one leptospiral species [3–5], suggesting that leptospiral infection, immunity, and transmission is more complex than previously thought. Additionally, the observed footprint of widespread genetic recombination among leptospiral strains [6, 7], provides evidence for a history of close interactions between replicating strains. For pathogenic leptospiral species that are primarily maintained and transmitted through animal hosts, frequent mixed infections may provide the mechanism to explain this genomic feature.

As part of a broader study on leptospirosis in an Ecuadorian region with high infection rates (research in progress under direction of Dr. Barragán and Dr. Pearson), we conducted a prospective urine analysis of dogs, as possible carriers, to explore the frequency and characteristics of mixed leptospiral infections. A total of 52 dogs have been tested in the largest project and 28 were positive. Of these animals, only 10 could be fully monitored for this study.

## Methods

### Sampling

The study was carried out in a rural coastal community in Manabí province where human and animal leptospirosis are endemic and, the population is primarily engaged in raising backyard farm animals [8]. The protocols used in this study were approved by the USFQ animal welfare committee (*Oficio*: *2021-010-extensión*). A total of 10 asymptomatic dogs were sampled over a period of 17 months (4 time points: October 2021, May 2022, October 2022, February 2023). Follow-up on some individual dogs was not possible due to death (unidentified disease or roadkill). Between 20 and 60 ml of urine samples were collected from each dog in a sterile container during spontaneous micturition. Bacterial cells in urine were concentrated using a 0.2 μm Millipore filter. Each filter was stored in RNA/DNA Shield (Zymo) in the dark and transported to the laboratory at room temperature.

#### *Leptospira* detection and species identification

Genetic material from the Millipore filters was extracted using a Qiagen DNAeasy kit. Detection of pathogenic *Leptospira* was performed using Taqman assays for *lipl32* [9] and SNP111 of the 16S rDNA gene [8]. A fragment of the *secY* gene (410 bp) was amplified in positive samples using the nested PCR assay described by Grillová and collaborators [10], with external primers [11]. Positive samples from which we could not obtain *secY* amplicons with the previous assay were analyzed with a nested PCR using the same external primers as Grillová et al. [10], and two novel internal sets of primers (S1 Fig). These novel primers were designed to specifically amplify sequences containing SNPs present in all species of the pathogenic P1 *Leptospira* clade (S2 Fig).

The sequences of designed primers were: Lepto_Ecu1_F: 5’-CCGCAAACGATCATTCAATGGTTATC-3’; Lepto_Ecu1_R: 5’AGAAGAGAAGTTCCACCAAACG-3’; Lepto_Ecu2_F: 5’-CGAACAGTGGGCGGGTT-3’; Lepto_Ecu2_R: 5’-GTTTCAAAGTCTCCAGCGCAAC-3’.

The *secY* gene fragments were sequenced using the Oxford Nanopore targeted sequencing platform which allows multiple and diverse sequences to be observed from a single PCR amplification. Consensus sequences were recovered using the Amplicon_sorter pipeline [v2023-06-19] without external references [12]. This pipeline searches for the sequences with the greatest similarity in base pairs and length to generate consensus sequences. If there are multiple sequences that differ in similarity and length, the algorithm generates multiple consensus sequences. These sequences were compared with representative genomes available in GenBank. MEGA11 [13] was used for phylogenetic analysis with the Maximum Likelihood method and Tamura-Nei model [14]. Our *secY* sequences can be found under bioproject accession number PRJNA1029631.

## Results

From the 10 dogs analyzed for this study (all positive for *Leptospira* DNA at different time points), there were 20 positive urine samples with eight dogs testing positive at multiple time points (Table 1).

**Table 1:** Detection and identification of *Leptospira* in dogs at more than one time points.

Sample	October 2021	May 2022	October 2022	February 2023
PG2			*Leptospira* spp.	*Leptospira* spp.
PG4			*Leptospira* spp.	*Leptospira* spp.
PG5			*Leptospira* spp.	*Leptospira* spp.
PG6		*Leptospira* spp.		*L*. *interrogans*
PG7		*L*. *interrogans*	*Leptospira* spp.	*Leptospira* spp.
PG8			*L*. *interrogans*	*Leptospira* spp.
PG9			*Leptospira* spp.	*Leptospira* spp.
P14G		*L*. *interrogans-L*.*santarosai*		
C7P2	*Leptospira* spp.	*L*. *interrogans-L*.*santarosai*	*Leptospira* spp.	
C3P1	*L*. *interrogans-L*.*santarosai*			

*Leptospira* species could be resolved in 6 samples: *Leptospira interrogans* was found in all of these samples and there was a mixed infection with *Leptospira santarosai* in three (C7P2, P14G and C3P1) (Table 1 and S1 Table). We were unable to identify *Leptospira* species in 14 follow-up samples due to poor DNA quality or low quantity.

**Fig 1:**
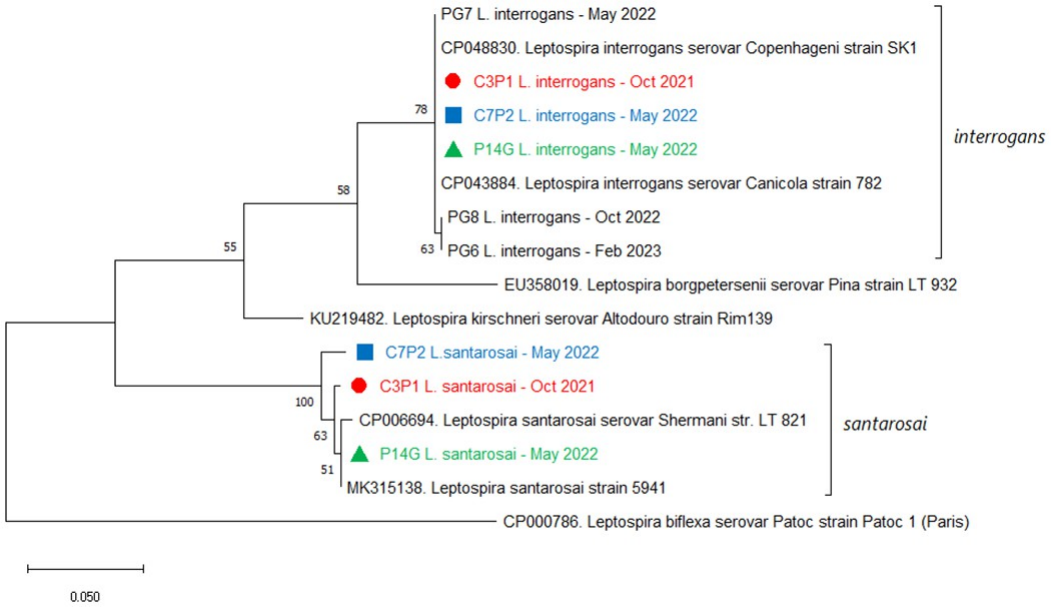
Phylogenetic analysis of the six sequenced samples. The tree with the highest log likelihood (-1375.62) is shown. The percentage of trees in which the associated taxa clustered together is shown next to the branches, which corresponds to 500 bootstrap pseudoreplicates. Colors indicate samples from the same dog at the same timepoint, showing the components of mixed infections.

The *L*. *interrogans secY* sequences obtained from 6 different dogs are identical, except for the same SNP in samples PG6 and PG8, while the three *L*. *santarosai secY* sequences showed polymorphisms (Fig 1).

## Discussion and conclusions

In this report, we show leptospiral co-infections in domestic animals, supporting previous findings in bats [3], and small rodents [4, 5]. Our results provide more evidence that mixed infections with more than one leptospiral strain in animal reservoirs (wild and domestic) may be more common than previously thought, and that the kidney may be the potential physical scenario for genetic recombination. This also suggests that tracking leptospiral transmission from domestic or wild animals to humans might be complex and require characterizing the infecting population rather than a single isolate or consensus sequence.

Although we are including few individuals in this study, we found 2 versions of the *secY* sequence in *L*. *interrogans* and 4 versions of the *secY* sequence in *L*. *santarosai* (Fig 1). This would suggest high strain diversity in this region. However, we must consider that there are only 6 dogs that we were able to sequence and that we are analyzing a partial region of a housekeeping gene. It would be interesting to follow up a larger number of individuals to look for strain turnover.

The newly designed PCR primers enabled us to amplify multiple *Leptospira* species from the same urine sample, enhancing our ability to detect a broader range of species simultaneously. However, we were unable to identify the species in 14 samples, possibly due to low DNA concentration or poor DNA quality.

In the largest project monitoring *Leptospira* infection in families and their domestic animals in this community, it was found that a pig, living in the same household as dog C3P1, also presented a co-infection of *L*. *interrogans* and *L*. *santarosai* species at the same time. However, the *L*. *santarosai secY* sequence isolated from the pig presents differences with the sequence isolated from the dog (S3 Fig).

Our findings, along with those of others [3–5], provide confirmatory evidence that mixed *Leptospira* infections occur in domestic and wild animals, and are likely to have played an important role in shaping the genomic content of *Leptospira* [7]. Mixed infections provide an ecological basis for genetic recombination between *Leptospira* species and strains. It has been suggested after an in-silico analysis that LPS genes (and many others) have undergone genetic recombination [7]. These genes are important for the diagnosis of leptospirosis and are also associated with protective immunity. Those previous results and ours suggest that there could be substantial implications for the development of vaccines and diagnostic tests as well as understanding the epidemiology and natural history of this genus.

## Supporting information

S1 Fig *secY* fragment amplified by nested PCR. Outer primers are labeled as Ahmed_F/Ahmed R (Ahmed *et al*., 2011). Inner primers amplifying the 410 bp fragment of the *secY* gene are labeled as follows: Picardeu_F/Picardeu_R (Grillová *et al*., 2020), Lepto_ECU1_F/ Lepto_ECU1_R, and Lepto_Ecu_2_F/ Lepto_Ecu_2_R (primers designed for this study).
(TIF)

S2 Fig Inner primers aligned with pathogenic *Leptospira* species belonging to the P1 clade. A. Picardeu_F/Picardeu_R (Grillová *et al*., 2020), B. Lepto_ECU1_F/ Lepto_ECU1_R, C. Lepto_Ecu_2_F/ Lepto_Ecu_2_R. *Leptospira interrogans* (GenBank: CPO48830.1) is used as reference. Note that the Picardeu primers have a perfect match to *L*. *interrogans* and primers designed for this study bind a higher diversity of pathogenic *leptospira* species.
(TIFF)

S3 Fig Phylogenetic analysis of the six sequenced samples from dogs and a sequenced sample from a pig (C3C11). The tree with the highest log likelihood (-1354.20) is shown. The percentage of trees in which the associated taxa clustered together is shown next to the branches, which corresponds to 500 bootstrap pseudoreplicates. Colors indicate samples from the same animal at the same timepoint, showing the components of mixed infections.
(TIF)

S1 Table *Leptospira* species identified by different primers sets, and number of reads recovered for each species. (XLSX)
