# Supplementary figures and images for: Mixed Leptospira infections in domestic animals from a rural community with high leptospirosis endemicity
Source: PLoS One. 2024 Oct 29;19(10):e0312556. doi: 10.1371/journal.pone.0312556 (PMC11521267; doi:10.1371/journal.pone.0312556)

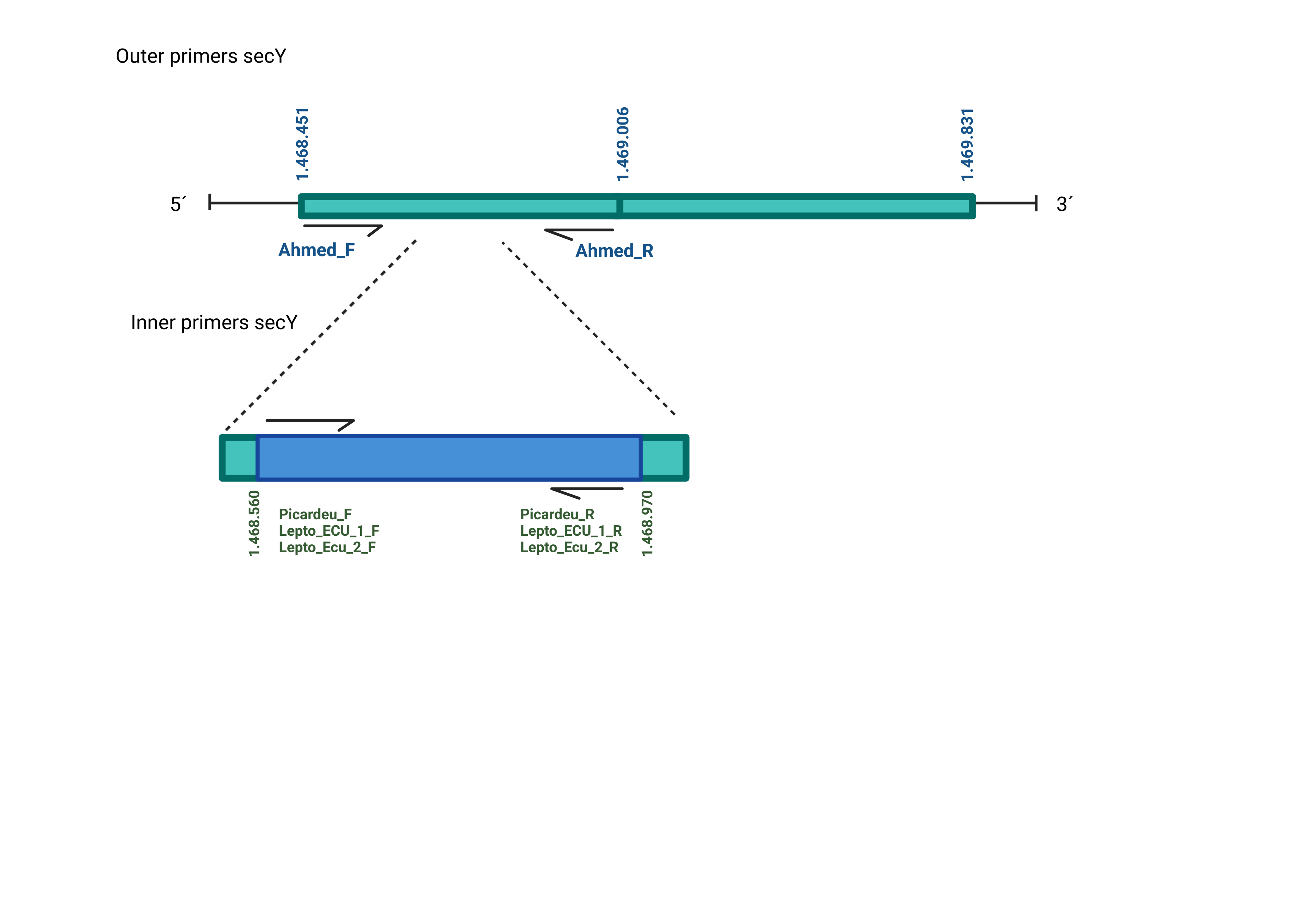

Supplement: S1 Fig — Outer primers are labeled as Ahmed_F/Ahmed R (Ahmed et al., 2011). Inner primers amplifying the 410 bp fragment of the secY gene are labeled as follows: Picardeu_F/Picardeu_R (Grillová et al., 2020), Lepto_ECU1_F/ Lepto_ECU1_R, and Lepto_Ecu_2_F/ Lepto_Ecu_2_R (primers designed for this study). (TIF) [file pone.0312556.s001.tif]

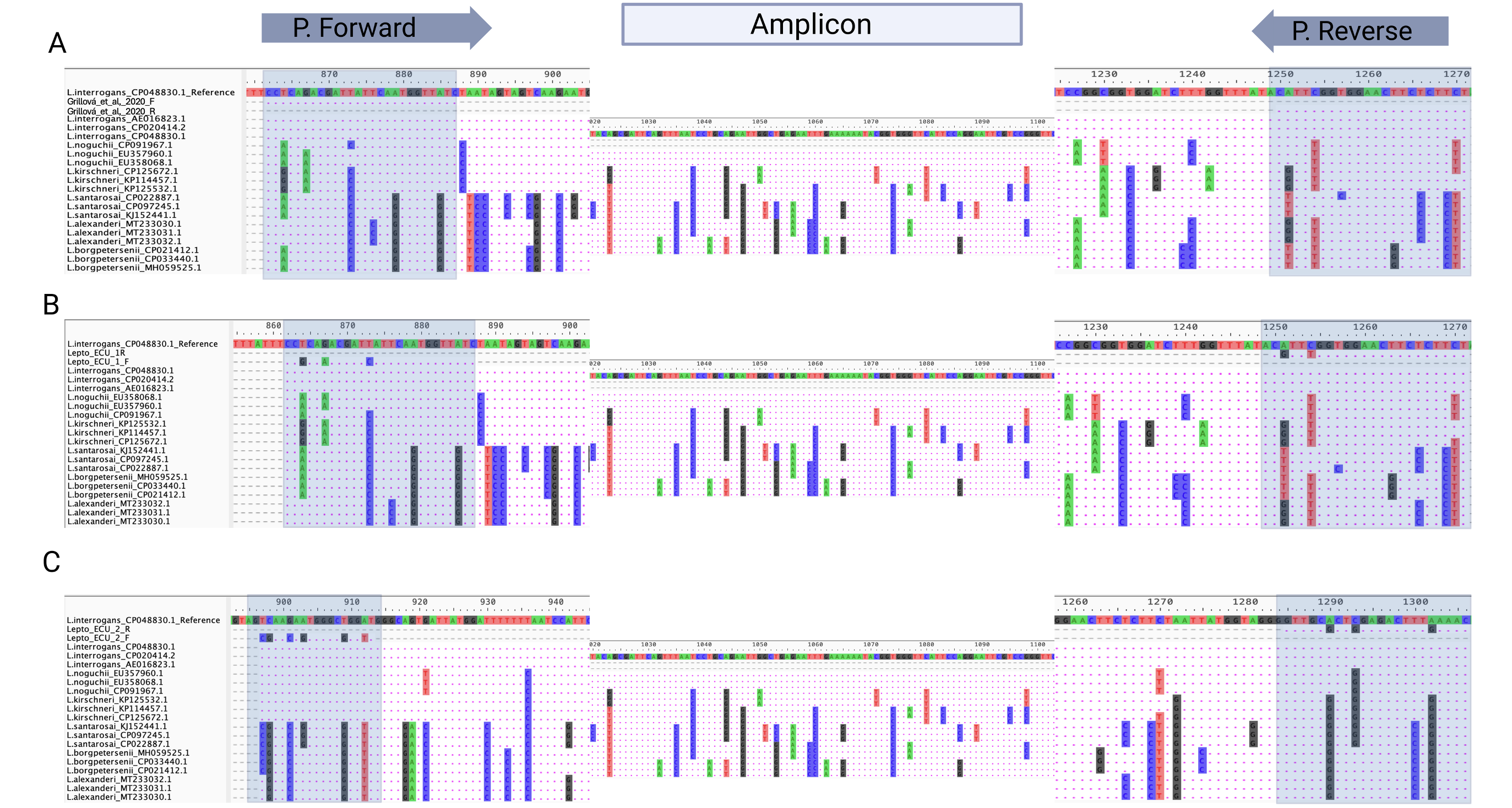

Supplement: S2 Fig — A. Picardeu_F/Picardeu_R (Grillová et al., 2020), B. Lepto_ECU1_F/ Lepto_ECU1_R, C. Lepto_Ecu_2_F/ Lepto_Ecu_2_R. Leptospira interrogans (GenBank: CPO48830.1) is used as reference. Note that the Picardeu primers have a perfect match to L. interrogans and primers designed for this study bind a higher diversity of pathogenic leptospira species. (TIFF) [file pone.0312556.s002.tiff]

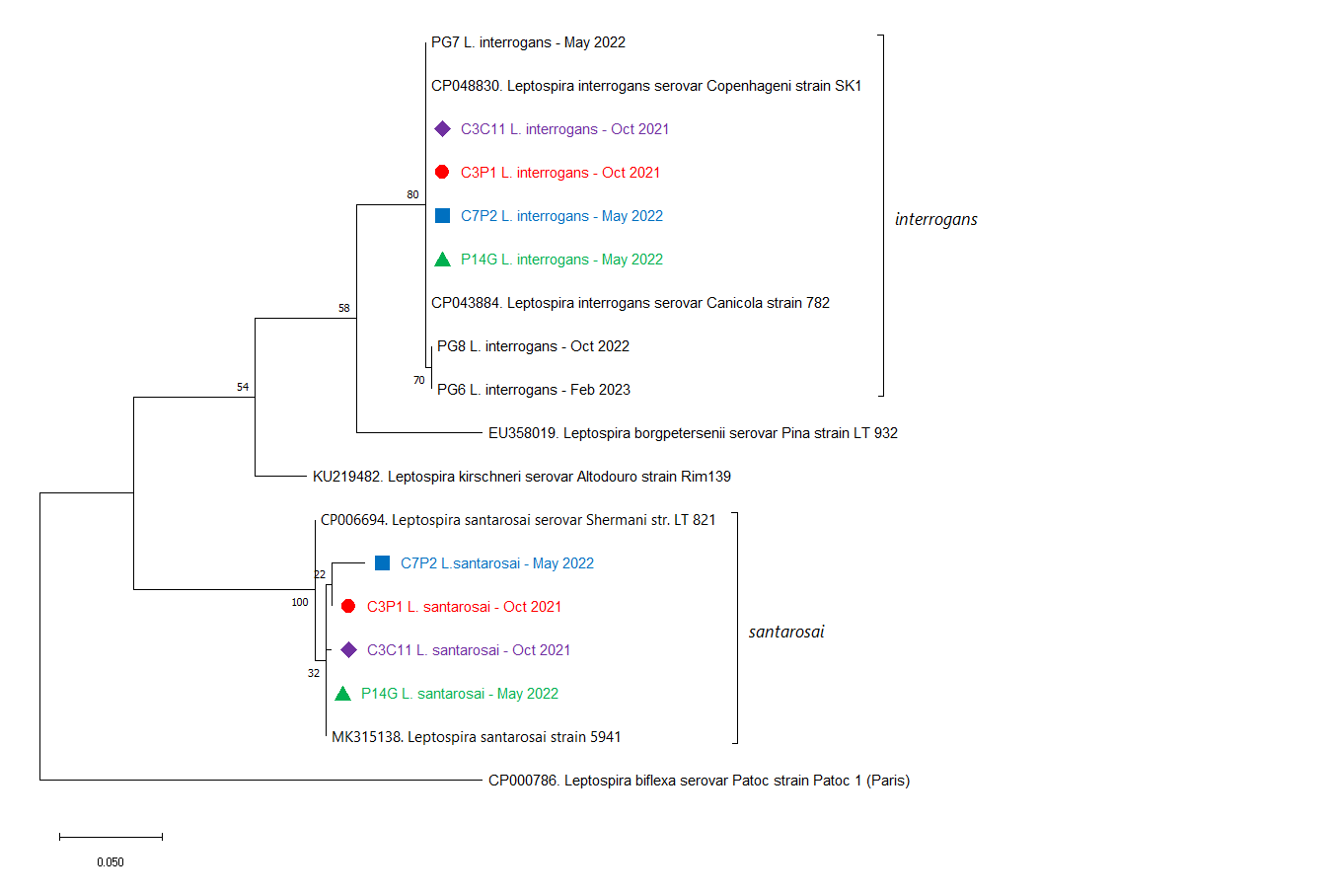

Supplement: S3 Fig — The tree with the highest log likelihood (-1354.20) is shown. The percentage of trees in which the associated taxa clustered together is shown next to the branches, which corresponds to 500 bootstrap pseudoreplicates. Colors indicate samples from the same animal at the same timepoint, showing the components of mixed infections. (TIF) [file pone.0312556.s003.tif]
